# Sodium Selenide Toxicity Is Mediated by O_2_-Dependent DNA Breaks

**DOI:** 10.1371/journal.pone.0036343

**Published:** 2012-05-07

**Authors:** Gérald Peyroche, Cosmin Saveanu, Marc Dauplais, Myriam Lazard, François Beuneu, Laurence Decourty, Christophe Malabat, Alain Jacquier, Sylvain Blanquet, Pierre Plateau

**Affiliations:** 1 Ecole polytechnique, Laboratoire de Biochimie, CNRS, Palaiseau, France; 2 Institut Pasteur, Unité de Génétique des Interactions Macromoléculaires, CNRS-URA2171, Paris, France; 3 Ecole polytechnique, Laboratoire des Solides irradiés, CNRS UMR7642-CEA, Palaiseau, France; Wayne State University, United States of America

## Abstract

Hydrogen selenide is a recurrent metabolite of selenium compounds. However, few experiments studied the direct link between this toxic agent and cell death. To address this question, we first screened a systematic collection of *Saccharomyces cerevisiae* haploid knockout strains for sensitivity to sodium selenide, a donor for hydrogen selenide (H_2_Se/HSe^−/^Se^2−^). Among the genes whose deletion caused hypresensitivity, homologous recombination and DNA damage checkpoint genes were over-represented, suggesting that DNA double-strand breaks are a dominant cause of hydrogen selenide toxicity. Consistent with this hypothesis, treatment of *S. cerevisiae* cells with sodium selenide triggered G2/M checkpoint activation and induced *in vivo* chromosome fragmentation. *In vitro*, sodium selenide directly induced DNA phosphodiester-bond breaks *via* an O_2_-dependent reaction. The reaction was inhibited by mannitol, a hydroxyl radical quencher, but not by superoxide dismutase or catalase, strongly suggesting the involvement of hydroxyl radicals and ruling out participations of superoxide anions or hydrogen peroxide. The ^•^OH signature could indeed be detected by electron spin resonance upon exposure of a solution of sodium selenide to O_2_. Finally we showed that, *in vivo*, toxicity strictly depended on the presence of O_2_. Therefore, by combining genome-wide and biochemical approaches, we demonstrated that, in yeast cells, hydrogen selenide induces toxic DNA breaks through an O_2_-dependent radical-based mechanism.

## Introduction

Selenium is mainly known as an essential micronutrient of many living species, including humans [1]. At high doses, selenium is poisonous [2]. In the recent years, selenium deserved considerable interest because of its possible protective effect against cancer at subtoxic doses [3]. Beyond its role in chemoprevention, subtoxic administration of selenium appears to also have a promising potential in cancer therapy [2], [4], [5], [6]. In all these medical applications, the gap between toxic and prophylactic or therapeutic doses is narrow. Although recent studies in a variety of model systems have increased our understanding of the anticarcinogenic mechanisms of selenium compounds, efforts still have to be made to complete our basic view of the underlying roles of selenium metabolites [3], [7].

Toxicity of selenium possibly combines several mechanisms such as redox cycling, DNA damage, glutathione (GSH) depletion or protein oxidation. Most of these mechanisms are linked to the pro-oxidant properties of some derivatives of selenium. In particular, it was early shown that, in the presence of thiols, selenite (SeO_3_^2−^) is reduced to hydrogen selenide, *i. e*. H_2_Se, HSe^−^ and Se^2−^, in chemical equilibrium. Upon oxidation by O_2_, hydrogen selenide produces elemental selenium and reactive oxygen species (ROS) including superoxide anions (O_2_^•−^) and hydrogen peroxide (H_2_O_2_) [8], [9], [10]. Production of hydroxyl radicals (^•^OH) was also proposed [11], [12], but, to our knowledge, experimental data in favor of such a production were never provided.

Although hydrogen selenide is believed to be a key player in the toxicity of inorganic selenium compounds [2], [13], its mechanism of action has hardly been experimentally studied. To fill this gap, we used *Saccharomyces cerevisiae* as a model [2]. Because this organism lacks the pathway for the specific incorporation of selenocysteine into proteins, interferences with the selenium incorporated in the active site of proteins are precluded.

In *S. cerevisiae*, several studies dealing with the toxicity of selenite, a precursor of hydrogen selenide, have been reported. A genome-wide transcriptome analysis revealed that selenite promoted the expression of most of the genes involved in the global oxidative stress response [14]. Moreover, glutathione reductase [15], [16], glutaredoxins [16], [17], [18] and Yap1p [16], a key transcriptional activator for the oxidative stress response, were shown to be involved in cell resistance to a selenite stress. All these studies indicate that selenite induces an oxidative stress *in vivo*. Other studies pinpoint involvement of selenium derivatives in genotoxic effects including base oxidations [15], [19], [20] and DNA breaks [15], [16], [21], [22].

To analyze the mechanism of toxicity of hydrogen selenide, we first screened a collection of gene deletion *S. cerevisiae* mutant strains for hypersensitivity to sodium selenide (Na_2_Se). We found a strong enrichment for homologous recombination (HR) and DNA damage checkpoint genes. Secondly, using flow cytometry, we showed that cells exposed to sodium selenide were blocked in the G2/M cell-cycle phase. Moreover, induction by Na_2_Se of DNA double-strand breaks (DSBs) *in vivo* was evidenced by pulse-field gel electrophoresis (PFGE) analysis. Next, using supercoiled DNA *in vitro*, we established that free radicals generated in solution by hydrogen selenide in the presence of dioxygen break phosphodiester bonds. Finally, consistent with the idea that the effect on DNA we describe *in vitro* might at least partly reflect what occurs *in vivo*, we observed that dioxygen potentiated the toxicity of hydrogen selenide against *S. cerevisiae* and that a mutant strain defective in HR displayed oxygen-dependent hypersensitivity to selenide.

## Materials and Methods

### Reagents

Mannitol, glutathione, sodium selenite, 2-morpholinoethanesulfonic acid (MES), 5,5′-dithiobis(2-nitrobenzoic) acid (DTNB), camptothecin (Cpt), dimethyl sulfoxyde (DMSO) and propidium iodide were from Sigma. H_2_O_2_ solution (30%, w/w) was from Merck. Catalase from beef liver, superoxide dismutase (SOD) from bovine erythrocytes, RNase A from bovine pancreas and SalI restriction enzyme were purchased from Roche Applied Science. Topoisomerase I from *Escherichia coli* was from New England BioLabs. 5-(diethoxyphosphoryl)-5-methyl-1-pyrroline *N*-oxide (DEPMPO) was obtained from Radical-Vision (Marseille, France). Sodium selenide (Na_2_Se) was from Alfa Aesar (Bischheim, France). In solution, Na_2_Se dissociates into 2 Na^+^ and Se^2−^. The latter ion partly protonates to give HSe^−^ and H_2_Se. According to the pK_a_ values of H_2_Se (3.9) and HSe^−^ (11.0), at pH 6.0 or 7.0, HSe^−^ is the most abundant species. For selenide stress challenge experiments, Na_2_Se powder was dissolved in a deoxygenated 50 mM MES buffer (pH 6.0), in an anaerobic glove box. In the case of flow cytometry, DNA breakage and electron spin resonance (ESR) experiments, dissolution was in a deoxygenated 100 mM potassium phosphate buffer (pH 7.0). Na_2_Se concentrations in solution were measured using the colorimetric assay described for hydrogen sulfide [23]. Briefly, samples (1 to 100 µl) were mixed with a 100 µM solution of DTNB in 1 ml final volume of MES buffer (50 mM, pH 6.0), prior to measurement of absorbance at 412 nm.

### Strains and Media

The *S. cerevisiae* strain BY4742 (MATα *his3*Δ*1 leu2*Δ*0 lys2*Δ*0 ura3*Δ*0*) and its isogenic *rad52*Δ mutant were from Euroscarf. The *RAD52* gene deletion was verified by multiple PCR reactions using primers outside and/or inside the integrated *kan*MX cassette. Rich YPD and YTD media contained 1% yeast extract (Difco), 2% glucose and either 1% Bacto-peptone (Difco) or 1% Bacto-tryptone (Difco), respectively. YTD medium was buffered at pH 6.0 by the addition of 50 mM MES-NaOH. Synthetic dextrose (SD) minimal medium contained 0.67% yeast nitrogen base (Difco), 50 mM MES-NaOH (pH 6.0), 2% glucose and 50 µg/l of each histidine, leucine, lysine and uracil.

To constitute a pool of *S. cerevisiae* mutants, cells from the systematic deletion collection made in strain BY4741 (MATa *his3*Δ*1 leu2*Δ*0 met15*Δ*0 ura3*Δ*0*) [24] were grown in individual wells in 96 deep-well plates at 30°C for 2 days in YPD medium. Pools of cells were obtained by mixing several cultures, divided in aliquots and stored at −80°C. The aliquots were thawed and allowed to recover in 800 ml of YTD medium for 9 h at 30°C, under shaking. Next, this preculture was used to inoculate three 1-liter cultures in YTD, at a final optical density (OD) of 0.2 at 650 nm. In two of the cultures, at t = 0, Na_2_Se was added at a final concentration of either 1 or 2 µM. The third culture was grown in the absence of Na_2_Se. All three cultures were grown aerobically at 30°C, under shaking. Every 4 h, the cultures were diluted to an OD_650_ of 0.2, and, in the case of the two subcultures challenged with selenide, fresh Na_2_Se was added back at final concentrations of 1 and 2 µM, respectively. At times 0, 16 h and 27 h, 30-ml aliquots were harvested and centrifuged to collect cells.

### Microarray Estimation of Growth Rates

The growth of individual strains in the pool of *S. cerevisiae* mutants was estimated from measurements done on barcode microarrays (see Methods S1 and Table S2 for a detailed protocol). To analyze the data, we calculated a relative fitness defect for each strain, defined as rf = log2(t_wt_^Se^/t_mut_^Se^–t_wt_/t_mut_+1), where t_wt_^Se^ (t_wt_) and t_mut_^Se^ (t_mut_) are the generation times of the wild-type and mutant strains in the presence (absence) of Na_2_Se, respectively (see Methods S2).

### Flow Cytometry Analysis

Wild-type BY4742 and its isogenic *rad52*Δ mutant were grown at 30°C in 100 ml YTD liquid medium. When the OD_650_ reached 0.5, the culture was divided into subcultures of 5 ml each. At t = 0, Na_2_Se dissolved in phosphate buffer or Cpt solubilized in DMSO were added and incubation was resumed. For the analysis of Cpt effect, DMSO was kept at a final concentration of 2% (v/v) in the growth medium to maintain Cpt solubility. We verified that, in the absence of Cpt, such a concentration of DMSO did not modify the cell-cycle distribution. After 2 h incubation, cells were harvested by centrifugation, washed in 1 ml of phosphate buffer saline (PBS) and fixed in 1 ml of cold 70% (v/v) ethanol. After 3 h at 37°C in 0.5 ml of PBS containing 1.5 mg/ml of RNase A, propidium iodide was added at a final concentration of 50 µg/ml and cells were incubated for 15 min at room temperature. Finally, cells were resuspended in 0.5 ml of PBS containing 5 µg/ml propidium iodide. Just before analysis with the FACS Calibur (BD Biosciences) cytometer, the solution was briefly sonicated to dissociate cell doublets. Analysis was performed with 60,000 cells for each condition.

### PFGE Analysis

BY4742 cells were grown at 30°C in 300 ml of SD medium. When the OD_650_ reached 1.0, the culture was divided in aliquots of 50 ml and sodium selenide was added to each aliquot at a final concentration of 0, 2.5, 5, 10 or 25 µM. After 1 h incubation at 30°C, cells were harvested by centrifugation, washed 3-times with 1 ml of 0.5 M EDTA (pH 8.0) and finally resuspended in 0.5 ml of 0.05 M EDTA. Chromosomes were prepared from 50 µl of the above cell suspension as described previously [25], except that agarose plugs were additionally incubated overnight at 37°C with 2 µg/mL of RNase A. Electrophoresis [25] was performed on a CHEF Mapper XA system (Bio-Rad) at 6 V.cm^−1^ for 28 h at 14°C. Separation was based on a two-state mode with angles of 60° and 180°, and a 60–120 s switch time ramp. After electrophoresis, the gel was stained with 50 µg/ml ethidium bromide for 30 min. Images were recorded on a Typhoon 9400 Imager (GE Healthcare) and bands intensities were determined with ImageJ software.

### Analysis of DNA Single-strand Breaking in Aerobic Conditions

pNOY102 plasmid [26] (10 µg/ml final concentration) was mixed on ice with potassium phosphate buffer (100 mM, pH 7.0) and with various components in different combinations involving sodium selenide (15 µM), sodium selenite (25 µM), glutathione (500 µM), mannitol (80 mM), catalase (50 units/ml), SOD (100 units/ml), Topo I (200 units/ml) and SalI (500 units/ml). Solutions were incubated 1 h at 37°C. Then, 150 mM of mannitol was added, and tubes were placed on ice. DNA molecules were loaded on a 0.7% (w/v) agarose gel in Tris-borate-EDTA buffer (45 mM Tris-borate (pH 8.3), 1 mM EDTA). After migration, the gel was stained with ethidium bromide.

### ESR Spectroscopy

DEPMPO spin trapper stock solution was prepared as previously described [27]. For analysis, solutions (400 µl) contained 250 mM potassium phosphate buffer (pH 7.0) and 120 mM DEPMPO. When added, mannitol, SOD and catalase were used at final concentrations of 150 mM, 100 units/ml and 500 units/ml, respectively. A freshly prepared solution of Na_2_Se was added to the sample just before analysis. After 5 min of incubation at 37°C, the sample was transferred in the ESR glass cell and spectra were immediately recorded. Control production of hydroxyl radicals by the Fenton reaction was obtained by mixing H_2_O_2_ (10 mM), FeSO_4_ (40 µM) and EDTA (80 µM). ESR experiments were performed with a Bruker EMX spectrometer operating at 9.7 GHz. An aqueous quartz flat-cell was used in a TE102 rectangular cavity. A microwave power of 20 mW and a field modulation of 2 gauss were used. For each sample, 6 successive scans from 338 to 358 mT were recorded (80 s per scan).

### Comparison of DNA Single-strand Breaking in Aerobic and Anaerobic Conditions (Glove Box Conditions)

Potassium phosphate (100 mM, pH 7.0) was prepared under aerobic conditions and then split into two aliquots. One of the aliquots was placed for 1 h under argon flow, before its transfer into an oxygen-free glove box (deoxygenated buffer). The other aliquot (oxygenated buffer) was transferred into a flask closed by an airtight rubber cap and then introduced into the glove box. Oxygenated buffer was sampled within the glove box with a Hamilton syringe, through the rubber cap.

To prepare an oxygen-free solution of DNA, pNOY102 solution was ethanol precipitated. The dry pellet was then introduced into the glove box and DNA was resuspended in the deoxygenated buffer. Under “anaerobic" conditions, oxygen-free DNA (20 µl at 1 µg/ml) was mixed with 40 µl of Na_2_Se at different concentrations and 40 µl of deoxygenated phosphate buffer. Under “aerobic" conditions, the DNA-Na_2_Se mixture was made similarly with 40 µl of oxygenated phosphate buffer so that, at the end, the soluble dioxygen concentration was 2.5-fold lower than in a solution prepared in open air. After incubation at room temperature (approximately 20°C) during 1 min, all reactions were quenched by the addition of mannitol at a 150 mM final concentration. Solutions were transferred outside the glove box for analysis by native agarose gel electrophoresis as described above.

### Analysis of Na_2_Se-dependent Lethality in the Presence or in the Absence of Dioxygen

All solutions were deoxygenated under nitrogen flow and equilibrated overnight in an oxygen-free glove box. BY4742 cells were grown aerobically in minimal SD medium and collected when the OD_650_ of the culture reached 0.5. For anaerobic conditions, a 30-ml aliquot was centrifuged, introduced in the glove box and resuspended in deoxygenated SD medium. To exhaust the remaining dioxygen, cells were incubated at 30°C for 30 min. Then, they were washed three times with MES buffer (50 mM, pH 6.0) and finally resuspended in MES buffer to obtain a OD_650_ of 0.1. After 5 min of incubation at 30°C with various concentrations of Na_2_Se (0–50 µM), cell suspensions were diluted 1000-fold in water and 150 µl of these dilutions were plated in duplicate onto YTD agar plates. Plates were placed inside an anaerobic jar containing a BioMérieux GENbox Anaer small bag. Next, the jar was removed from the glove box to a 30°C incubator. After 5 days, the colonies were counted. For aerobic conditions, the experiment was driven in the same way out of the glove box and with oxygenated solutions. The colonies were counted after a 2-day incubation at 30°C.

## Results

### A Genetic Screen Highlights a Key Role of Recombinational Repair and Checkpoint Genes in Resistance to Sodium Selenide

To identify cellular processes involved in *S. cerevisiae* resistance to hydrogen selenide, we analyzed the sensitivity of a collection of approximately 5000 isogenic haploid knockout mutants to sodium selenide, a donor of hydrogen selenide [24]. Because oxidation of hydrogen selenide is rapid in an oxygenated solution (half-life <2 min in rich YTD medium at 30°C and pH 6.0), the selenide stress challenge was performed by renewing sodium selenide addition every 4 h. Two experiments were performed with 1 or 2 µM sodium selenide final concentrations, respectively. At such concentrations, the doubling time of the wild-type strain was increased by 30–40%. Cells were collected after 16 and 27 h. DNA barcode regions of the various strains were amplified by PCR and labeled with Cy3 or Cy5 fluorophores (Figure S1). Hybridization of these PCR products on Agilent barcode microarrays was used to derive relative fitness defect estimates for individual mutants (see Materials and Methods).

As shown in Table S1, the results were similar for the two concentrations of Na_2_Se used (1 and 2 µM). Thus, the two sets of data were fused. The resulting distribution of the fitness values was asymmetric with a long tail on the negative side, as expected from a selective effect of sodium selenide treatment on a subset of the mutants from the collection (Figure S2A). Because of this asymmetry and of the broader-than-Gaussian shape of the density distribution, a z-type statistics could not be used to analyze the results. Instead, we examined the ranks of the values (see Table 1 for the annotated list of the 30 most selenide-sensitive deletion strains, and Table S1 for the full list).

**Table 1:** Ranking of genes (1 to 30) based on relative fitness defects calculated for deletion strains after treatment with sodium selenide.

rank	rf^a^	gene name	description/function	DSB^b^	Cpt^c^	γ-rays^d^	GSH^e^
1	−1.75	*GSH1*	γ-Glutamylcysteine synthetase				+
2	−1.70	*RAD57*	Repair of DSBs in DNA	+	+	+	
3	−1.68	*GLR1*	Glutathione reductase				+
4	−1.67	*HOM6*	Homoserine dehydrogenase				
5	−1.62	*YAF9*	Chromatin remodeling, DNA repair	+			
6	−1.59	*RAD52*	Repair of DSBs in DNA	+	+		
7	−1.47	*GRX1*	Glutaredoxin 1				+
8	−1.42	*DDC1*	DNA damage checkpoint	+	#	+	
9	−1.39	*RAD9*	DNA damage checkpoint	+		+	
10	−1.37	*RAD24*	DNA damage checkpoint	+	#	+	
11	−1.37	*NPP1*	Nucleotide diphosphatase				
12	−1.29	*YMR031W-A*	Dubious ORF overlapping *YMR031C* (putative mitochondrial protein of unknown function)				
13	−1.28	*RAD17*	DNA damage checkpoint	+	#	+	
14	−1.25	*YMR221C*	Putative mitochondrial protein of unknown function				
15	−1.23	*DCC1*	Sister chromatid cohesion establishment	+	#		
16	−1.21	*MUP1*	High affinity methionine permease				
17	−1.20	*RAD59*	Repair of DSBs in DNA	+	+	+	
18	−1.17	*PRB1*	Vacuolar proteinase B				
19	−1.15	*YBR099C*	Dubious ORF overlapping *MMS4*		+		
20	−1.14	*RRT12*	Probable subtilisin-family protease				
21	−1.13	*FET4*	Low-affinity Fe(II) transporter of the plasma membrane				
22	−1.12	*ATG15*	Lipase required for autophagy				
23	−1.10	*DUN1*	DNA damage checkpoint	+			
24	−1.09	*YNL324W*	Dubious ORF overlapping *FIG4*				
25	−1.08	*SWR1*	Chromatin remodeling				
26	−1.04	*MUS81*	Subunit of the Mms4p-Mus81p endonuclease involved in DNA repair	+	+	+	
27	−1.03	*RAD51*	Repair of DSBs in DNA	+	+		
28	−1.03	*YCR050C*	Protein of unknown function				
29	−1.02	*VAC14*	Synthesis of phosphatidylinositol 3,5-bisphosphate				
30	−1.02	*RPS21B*	Protein of the small ribosomal subunit				

a rf: relative fitness defect.

b DSB : genes of the list encoding proteins implicated in cellular response to DSBs (HR and DNA damage checkpoint) are labeled with +.

c Cpt: genes of the list having a low rank in a screen with Cpt, as reported by Hillenmeyer *et al*. [45]. Genes ranking under 30 are labeled with +, genes ranking between 31 and 150 are labeled with #.

d γ-rays: genes ranking under 30 in a γ-rays screen, as reported by Game *et al*. [30] (data regarding higher ranks are no more available on the referenced web site).

e GSH: the designated proteins are involved in glutathione homeostasis.

Two criteria validated the screen *a posteriori*. First, we found similar sensitivities to sodium selenide when two independent disruptions of a same gene were available and compared. This was the case with overlapping ORFs (Figure S2B, see also, for example, *MMS4* and *YBR099C*, or *FIG4* and *YNL324W* in Table 1). Secondly, deletion mutants for genes coding for distinct subunits of a same protein complex often ranked similarly. This was the case of the Rad55p-Rad57p complex (rank 2 for *RAD57* and 45 for *RAD55*), the Mus81p-Mms4p complex (rank 26 for *MUS81* and 52 for *MMS4*) and the Vac14p-Fig4p complex (rank 29 for *VAC14* and 32 for *FIG4*). Notably, out of the 9 nonessential genes encoding subunits of the Swr1 complex, 7 (*SWR1, YAF9*, *SWC5*, *ARP6*, *SWC3*, *VPS71*, *VPS72*) belonged to the 100 first ranked genes.

**Figure 1:**
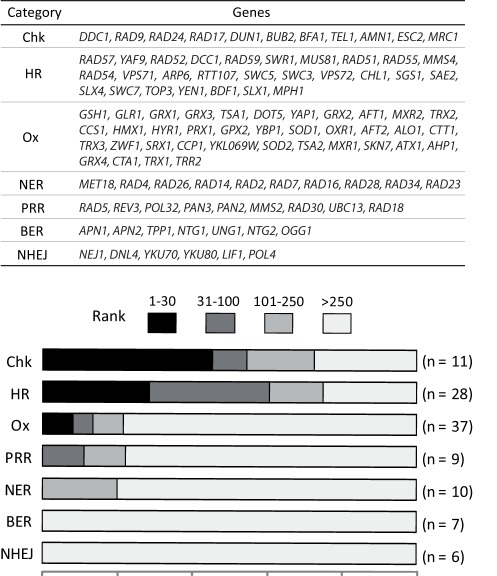
Sodium selenide sensitivity distribution of mutants for genes involved in DNA damage and oxidative stress responses. In the upper panel, 7 gene categories were defined. Selected genes are given in ranking order. Chk, DNA-damage checkpoint category; HR, homologous recombination; Ox, oxidative stress response; NER, nucleotide excision repair; PRR, post-replicative repair; BER, base excision repair; NHEJ, non-homologous end joining. In the lower panel, the 7 categories are presented with black, dark-gray, light-gray and white bars proportional to the percentages of genes ranking under 30, between positions 31 and 100, between 101 and 250 and over 250, respectively. The numbers (n) of genes retained in each category are shown at the right side. Essential genes of interest (such as *RAD53*, *CDC9*, *MEC1*, *TRR1*,…) were ignored because of the lack of the corresponding mutants in the collection. Two genes (*RAD6* and *MEC3*) were not included in the above categories because they belonged to those for which we could not obtain more than two sets of data (see Methods S2). Eight genes belonging to more than one of the above 7 metabolic pathways were discarded from the analysis. They include *SRS2*, *RAD50* and *XRS2* (HR and NHEJ), *DOT1* (HR, PRR and NER), *BRE1* (HR and Chk), *RAD27* (NHEJ and BER), *RAD10* and *RAD1* (NHEJ, PRR and NER). Actually, none of these genes ranked below 100 in Table S1. Three of them (*SRS2*, *RAD10*, *RAD1*) were between positions 101 and 250. The other ones ranked over 250. Whether these genes were taken into account or not did not significantly influence the histogram (data not shown).

Strikingly, our screen revealed that 29% of the 28 genes belonging to the HR pathway ranked in the first 30 (with a p-value of 4.2·10^−14^), and 75% under 250 (p = 7.4·10^−23^) (Figure 1). In particular, all genes encoding proteins involved in synapsis (Rad51p, Rad52p, Rad54p, Rad55p and Rad57p) ranked below 53 (p = 3.4·10^−10^) with a median rank of 27. Similarly, 73% of the 11 genes associated with checkpoint activation were found in the first 250 ranks (p = 2.3·10^−11^), while 45% ranked in the first 30 (p = 8.5·10^−11^), with particularly low ranks for *DDC1*, *RAD9*, *RAD24*, *RAD17* and *DUN1* (8, 9, 10, 13 and 23, respectively). Among the genes with a ranking lower than 100, were also found the 2 genes encoding the Mus81p-Mms4p complex involved in the resolution of Holliday junctions [28] and, as already mentioned, 7 genes encoding subunits of the Swr1 complex responsible for chromatin remodeling during DSB repair [29]. These results establish that HR and checkpoint genes are important for cell resistance to selenide and suggest that genotoxicity resulting from DSB formation is a key component of selenide toxicity. In favor of DSB occurrence *in vivo* upon sodium selenide treatment, the results of our genetic screen resemble other results obtained using ionizing radiation or Cpt, two agents known to induce DSBs [30], [31] (Table 1).

None of the genes encoding the Mre11p-Rad50p-Xrs2p (MRX) complex and the nuclease Sae2p, which cooperate in resection initiation, emerged in the first 200 ranks. Possibly, as already observed for the repair of multiple DSBs induced by HO endonuclease [32], MRX-dependent resection initiation plays a facultative role in the DNA repair after sodium selenide treatment. Moreover, none of the base excision repair (BER, 7 genes) and non-homologous end joining (NHEJ) genes (6 genes) were in the first 250 ranks (median ranks of 1774 and 2505, respectively) (Figure 1). In between, nucleotide excision repair (NER, 10 genes) and post-replicative repair (PRR, 9 genes) showed an intermediate distribution, with (i) no gene ranking under 30, (ii) 20% of NER genes and 22% of PRR genes between positions 31 and 250, and (iii) overall median ranks of 1393 (NER) and 1435 (PRR). Thus, after a sodium selenide challenge, HR dominates other repair pathways in cell survival. This advocates once more for the importance of DSBs in the toxicity of selenide.

The genes of γ-glutamylcysteine synthethase, glutathione reductase and glutaredoxin 1 belong to the first 10 ranked genes. That of glutaredoxin 3 and *TSA1*, the gene of a thioredoxin peroxidase, have ranks 31 and 65, respectively (Table 1). The low ranks of these genes, which are involved in the oxidative stress pathway, indicates that redox potential is also an important factor in the cell resistance to sodium selenide. Indeed, reduced gluthatione, glutaredoxin and thioredoxin are antioxidants preventing damages caused by reactive oxygen species such as free radicals and peroxides. These thiol-containing agents can also be involved in the recycling of protein disulfides or protein-selenotrisulfides produced in the course of H_2_Se redox cycling [7].

### G2/M Cell Cyle Checkpoint is Activated by Sodium Selenide Treatment

The genome-wide screen suggested the induction of DSBs by sodium selenide. Consequently, a cell-cycle arrest should be observed. Indeed, the cell-cycle progression of cells exposed to genotoxic agents can be perturbed in two ways, depending on the effect on the replication process. On the one hand, DNA damages which block the replication forks (such as some base oxidations, base methylations, intra- or inter-strand cross-links…) trigger the activation of an intra-S checkpoint and the S-phase is prolonged until DNA repair occurs [33]. On the other hand, in the presence of SSBs or DSBs, replication forks occasionally collapse. However, replication fork block does not occur, and checkpoint activation does not interfere with S-phase progression. As a result, cell-cycle blockage and DNA repair are delayed to G2 [33].

**Figure 2:**
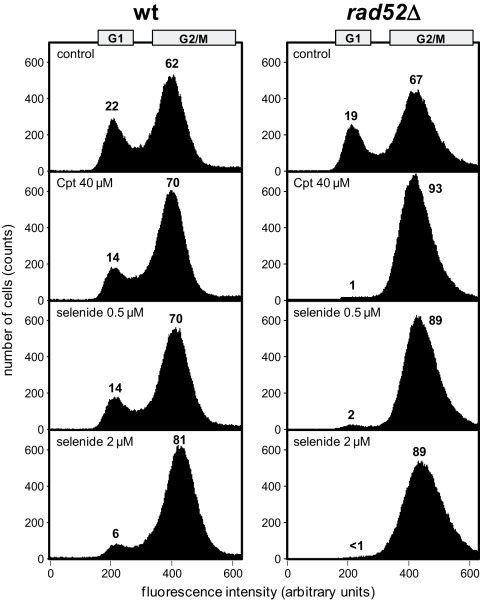
Analysis of cell-cycle phases distribution in asynchronous cultures of wild-type (wt) and *rad52* Δ **strains.** Before analysis by flow cytometry, cells were grown for 2 h in YTD in the presence of the indicated concentrations of Cpt or sodium selenide. The histogram shows the number of cells (counts) corresponding to each fluorescence intensity (in arbitrary units). The percentages of G1 and G2/M subpopulations are indicated above the corresponding peaks. The boxes at the top of the panels indicate the fluorescence intensity intervals chosen to calculate G1 and G2/M cell populations.

To investigate whether selenide induced a cell-cycle arrest, we followed cell-cycle progression of the wild-type strain and of its isogenic *rad52*Δ mutant. *RAD52* inactivation is known to impair the HR pathway [34]. Cpt, which induces DNA breaks, was used as control. Cell-cycle progression of cells incubated during 2 h with 40 µM Cpt or with 0.5 or 2 µM Na_2_Se was monitored using flow cytometry (Figure 2). In the absence of toxic agent, both wild-type and *rad52*Δ strains were predominantly in the G2/M phase (at least 62% of the cells possessed a 2 n DNA content). As expected, Cpt treatment of the wild-type strain caused an increase in G2/M percentage (+8%) paralleled by a comparable decrease in G1 percentage. The block in G2/M was amplified upon *RAD52* inactivation (+26% of G2/M cells) and appeared to be more time-stable, as indicated by near complete disappearance of G1 cells.

When the wild-type strain was exposed to sodium selenide, the G2/M population increased. At the highest concentration of Na_2_Se (2 µM), the effect was dramatic. The G2/M population increased by 19% whereas the G1 population dropped to 6%, and quasi-complete synchronization of the cell population occurred. Importantly, at the lowest used concentration of Na_2_Se (0.5 µM), the increase in the G2/M percentage was nearly three times higher with the *rad52*Δ strain than with the wild-type strain (22% and 8%, respectively), indicating that selenide-induced cell-cycle blockage is amplified by *RAD52* inactivation. The similarity of the effects of Cpt and sodium selenide on G2/M checkpoint activation, both with the wild-type and *rad52*Δ strains, is consistent with DSB being the major DNA damage caused by selenide.

### Sodium Selenide Induces Double-strand Breaks *in vivo*

**Figure 3:**
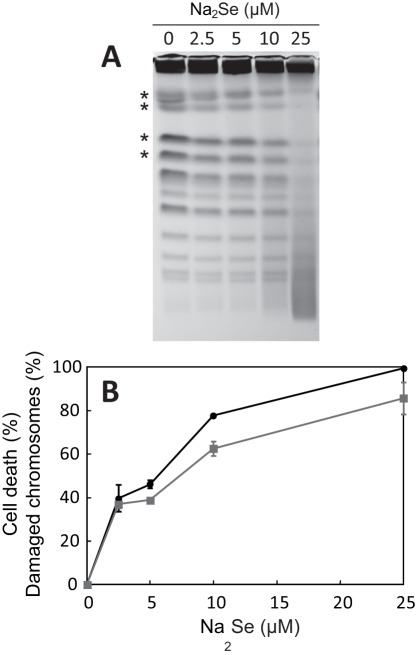
PFGE analysis of the effect of sodium selenide on DNA integrity *in vivo*. Exponentially growing *S. cerevisiae* cells were incubated for 1 h in the presence of Na_2_Se at the indicated concentrations. (A) Chromosomal DNA was extracted, separated by PFGE and stained with ethidium bromide. (B) Cell survival was measured in parallel by counting colony-forming units. Aliquots of the cultures were diluted 10,000-fold in water and 200 µl of these dilutions were plated in duplicate onto YTD agar plates. Results are expressed as cell death percentages (mean value and range of the duplicate measurements) relative to the number of survivors in the culture without sodium selenide (circles). To quantify the levels of DNA breaks in cells grown in the presence of sodium selenide in solution, the intensities of the four bands corresponding to the four largest chromosomes (indicated by stars in panel A) were determined by gel optical scanning. The intensities of the bands in the presence of Na_2_Se were expressed as percentages of the corresponding intensities in the control without Na_2_Se. Next, in each Na_2_Se condition, the four obtained percentage values were averaged and a standard deviation was calculated (squares).

These results prompted us to determine if DSBs were indeed produced in cells treated with sodium selenide. *S. cerevisiae* cells grown in minimal medium were incubated in the absence or in the presence of increasing concentrations of Na_2_Se. After 1 h of incubation, cells were included into agarose plugs and their chromosomes were analyzed by PFGE (Figure 3A). Cell survival after the treatment was also measured (Figure 3B).

In the absence of sodium selenide, yeast chromosomes migrated as discrete bands. In the presence of sodium selenide, the intensities of the chromosome bands decreased, whereas smears corresponding to shorter DNA fragments accumulated. This DNA fragmentation was accompanied by a decrease in cell viability. To estimate the level of damaged chromosomes in each growth condition, we quantified the bands corresponding to the largest chromosomes (Figure 3B). For smaller chromosomes, bands could not be distinguished from the breakage products of the longer chromosomes. Comparison of the two curves shown in Figure 3B indicates that the increase in chromosome damage was very similar to the variation of cell death. We conclude that, in the presence of Na_2_Se in the culture medium, DSBs are produced and that the rate of DSB formation tightly correlates with the rate of cell death.

### Selenide, but not Selenite, Breaks Phosphodiester Bonds *in vitro*

**Figure 4:**
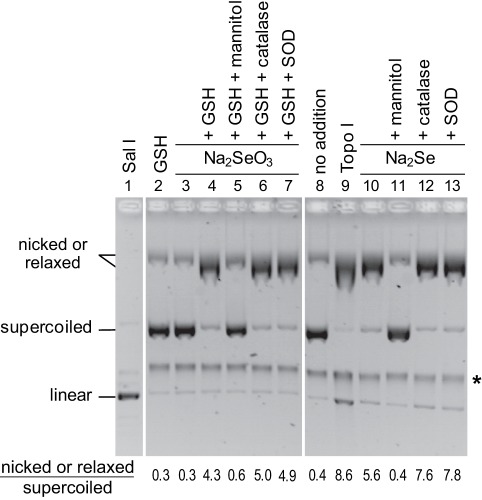
Effect of sodium selenide on DNA integrity *in vitro*. Purified pNOY102 plasmid DNA was submitted to native gel electrophoresis after incubation during 1 h at 37°C with different combinations of the indicated compounds: 15 µM Na_2_Se, 25 µM sodium selenite (Na_2_SeO_3_), 500 µM glutathione (GSH), 200 units/ml SOD, 80 mM mannitol, 50 units/ml catalase, 200 units/ml topoisomerase I (Topo I) or 500 units/ml SalI. The different topomers, as detected after ethidium bromide staining, are indicated on the left. The star on the right side of the figure points a contaminant systematically present in our pNOY102 preparations. The numbers at the bottom of the figure show the ratios between nicked and supercoiled DNA band intensities. With topoisomerase, the ratio is between relaxed and supercoiled DNA.

The DNA breaks we observed *in vivo* can be caused by hydrogen selenide either directly or indirectly. To help distinguish between these two possibilities, we asked whether addition of Na_2_Se broke DNA in a minimal system consisting of supercoiled DNA in an oxygenated phosphate buffer. In this system, conversion of the supercoiled plasmid into its nicked form which has a lower electrophoretic mobility allows to detect SSBs. Electrophoresis conditions were set up to separate supercoiled, nicked and linear pNOY102 plasmid DNAs (compare lanes 1, 8 and 9 in Figure 4).

When incubated for 1 h at 37°C in the presence of fresh Na_2_Se (15 µM), the plasmid was almost entirely converted into its nicked form (compare lanes 10 and 8 in Figure 4). In contrast, sodium selenite did not break DNA (lane 2) unless it was mixed with glutathione (lane 4), a condition known to convert selenite into hydrogen selenide [13], [35]. Thus, we conclude that selenide is sufficient to break phosphodiester bonds, whereas selenite alone has no detectable effect.

### Free Radical Production is Required for Selenide to Nick DNA

To determine whether ROS were involved in selenide-induced DNA breakage, the above experiment was repeated in the presence of a radical quencher or of detoxication enzymes. Before addition of sodium selenide or sodium selenite, the plasmid was mixed with either SOD (which converts superoxide anions O_2_^•−^ into H_2_O_2_ and O_2_), catalase (which converts H_2_O_2_ into H_2_O plus O_2_) or mannitol (a quencher of various radicals including ^•^OH, but, importantly, not O_2_^•-^). The electrophoretic profiles of plasmid DNA samples recorded after treatment with either sodium selenide or sodium selenite plus glutathione were not modified by the presence of catalase or SOD (compare lanes 6 and 7 to lane 4, and lanes 12 and 13 to lane 10 in Figure 4). On the other hand, in the presence of mannitol, the profiles remained similar to that observed when sodium selenide was omitted (compare lanes 4 and 11 to lane 8 in Figure 4). These data indicate that one or several free radicals, with the exception of O_2_^•−^, are involved in the selenide-induced DNA damaging reaction.

ESR spectroscopy was used to monitor the production of radicals from sodium selenide. Reaction of the spin trapper DEPMPO with ^•^OH radicals leads to an oxidized stable form (DEPMPO-OH) which has a characteristic 8-peak spectrum [36]. Superoxide anions also react with DEPMPO, but the resulting modified spin trapper (DEPMPO-OOH) displays a clearly distinct ESR spectrum [36].

**Figure 5:**
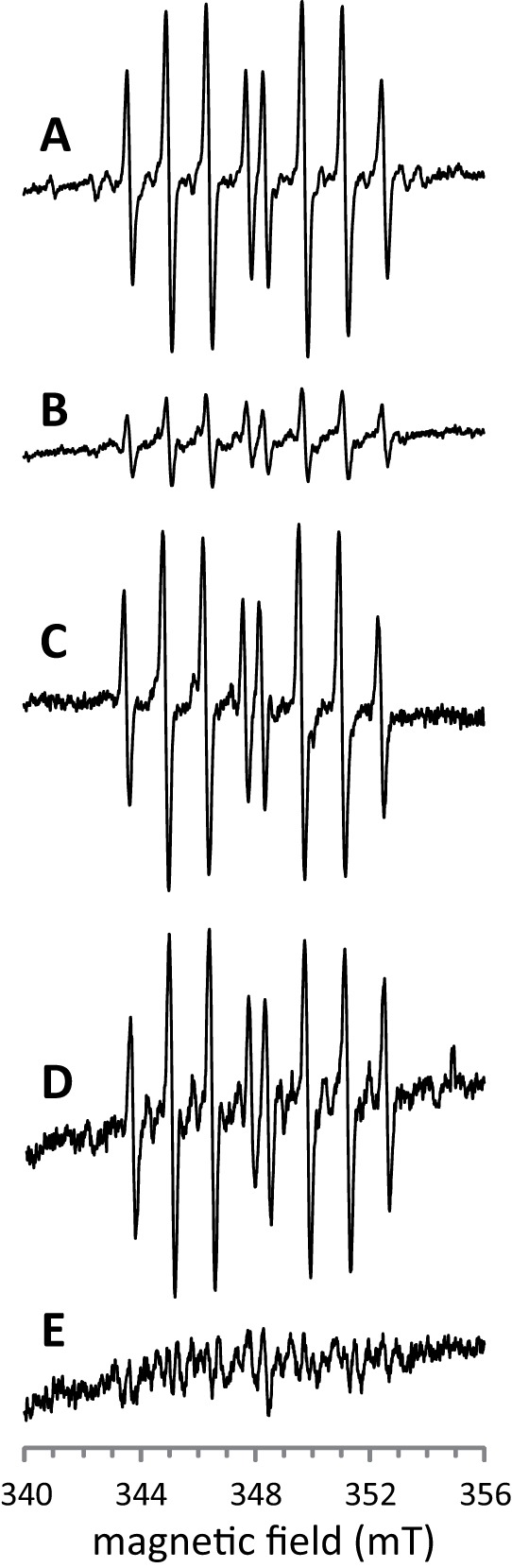
ESR analysis of the oxidation of sodium selenide in the presence of dioxygen. Before measurements, DEPMPO spin trapper (at a final concentration of 120 mM in 400 µl-samples) was incubated for 5 min at 37°C with the following compounds. (**A**) 10 mM H_2_O_2_+40 µM Fe(II)-EDTA+SOD (40 units). (**B**) 10 mM H_2_O_2_+40 µM Fe(II)-EDTA+150 mM mannitol+SOD (40 units). (**C**) 100 µM Na_2_Se. (**D**) 100 µM Na_2_Se+SOD (40 units)+catalase (200 units). (**E**) 100 µM Na_2_Se+150 mM mannitol+SOD (40 units)+catalase (200 units). The ordinate scale is the same for spectra (**A**) and (**B**) and for spectra (**C**), (**D**) and (**E**).

We first carried out a control ESR analysis after incubation of DEPMPO with H_2_O_2_ and Fe(II)-EDTA. This mixture is expected to lead to ^•^OH production through the Fenton reaction. Consistent with ^•^OH formation in this control experiment, the spectrum of DEPMPO-OH was observed (Figure 5A). In the presence of mannitol (150 mM) in the mixture, the magnitude of the spectrum was strongly reduced (Figure 5B). This confirmed that the spectrum in Figure 5A indeed reflected ^•^OH production.

To analyze radical production in the presence of selenide, DEPMPO was incubated in the presence of Na_2_Se (100 µM). The eight-peak spectrum characteristic of DEPMPO-OH was obtained (Figure 5C). Because DEPMPO-OOH can slowly convert to DEPMPO-OH [36], we performed the same experiment in the presence of SOD to make sure that the DEPMPO-OH spectrum did not arise from a conversion of DEPMPO-OOH to DEPMPO-OH. We also added catalase to the reaction mixture to preclude formation of hydroxyl radicals from SOD-produced H_2_O_2_. Indeed, parasitic generation of ^•^OH might have occurred in the case of contamination of the solution by trace amounts of transition metals. Under these conditions (SOD and catalase), the eight-peak spectrum of DEPMPO-OH was again obtained (Figure 5D). Similarly to what we observed in the Fenton reaction control experiment, the amplitude of the spectrum strongly decreased when the mixture of DEPMPO and Na_2_Se was supplemented with mannitol (Figure 5E). Thus, we conclude that the reaction of selenide with dioxygen produces free radicals, likely ^•^OH radicals, and that if superoxide ions were produced, their concentration remained below the detection threshold.

### DNA Damage by Sodium Selenide Requires the Presence of Dioxygen

To establish whether the DNA-damaging reaction required the presence of dioxygen, we compared the rates of selenide-induced SSB formation under aerobic and anaerobic conditions, *in vitro*. In the experiment shown in Figure 5A, increasing amounts of Na_2_Se were added to the pNOY102 plasmid DNA solution. Incubations were performed inside a glove box with dioxygen partial pressure continuously lower than 5 ppm. DNA solutions were prepared in the glove box in either a deoxygenated buffer (anaerobic conditions) or an oxygenated one (aerobic conditions). The reaction was initiated by mixing the DNA solutions with Na_2_Se, and quenched after 1 min by addition of 150 mM mannitol.

**Figure 6:**
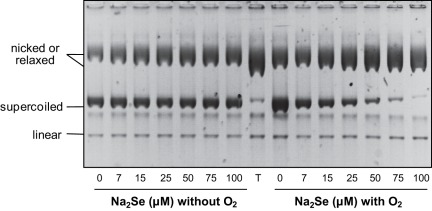
Effect of dioxygen on the *in vitro* Na_2_Se-induced DNA nicking. Purified pNOY102 plasmid DNA was incubated for 1 min at 20°C with the indicated concentrations of sodium selenide in either a deoxygenated (left) or an oxygenated buffer (right). The reaction was quenched by the addition of mannitol. The different topomers were then separated by native gel electrophoresis and detected by ethidium bromide staining. Lane T corresponds to a topoisomerase I-relaxed DNA sample.

In the oxygenated buffer, in agreement with the results presented in Figure 4, addition of increasing amounts of sodium selenide caused progressive nicking of supercoiled DNA, as shown by the decreasing intensity of the corresponding gel band (Figure 6, right side). The nicking of plasmid DNA was nearly complete in the presence of 0.1 mM Na_2_Se. In contrast, supercoiled DNA remained unmodified by sodium selenide in deoxygenated buffer (Figure 6, left side). We conclude that selenide-induced DNA breakage strictly requires the presence of dioxygen.

### Dioxygen in the Culture Medium Potentiates the Toxicity of Sodium Selenide *in vivo*

**Figure 7:**
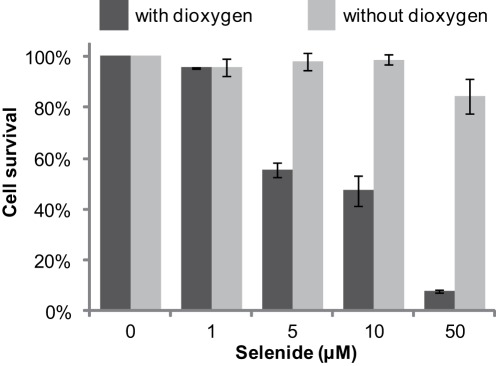
Effect of dioxygen on *S. cerevisiae* Na_2_Se-induced lethality. BY4742 cells were exposed for 5 min to the indicated concentrations of sodium selenide, and plated on rich medium. Colonies were counted after 2 days (in the presence of O_2_, black bars) or 5 days (in the absence of O_2_, gray bars) of incubation at 30°C. Shown in ordinate are the percentages of survival relative to controls in the absence of Na_2_Se. Ranges were deduced from duplicate experiments.

Because selenide requires dioxygen to nick DNA i*n vitro,* we asked whether dioxygen was necessary for selenide to cause death of yeast cells. The wild-type *S. cerevisiae* strain was exposed to various concentrations of Na_2_Se (0–50 µM) for 5 min, under aerobic or anaerobic conditions. To estimate short-term viability, cells were plated on rich medium just after the treatment and their ability to form colonies was determined. As expected, in the presence of dioxygen, Na_2_Se concentrations higher than 5 µM induced a significant loss of viability (Figure 7). When cells were maintained in strict anaerobic conditions, cell death was no longer observed in the presence of sodium selenide. This experiment establishes that selenide toxicity indeed implies an oxygen-dependent mechanism.

## Discussion

### Free Radicals are Produced During Oxidation of Hydrogen Selenide by Dioxygen

This study provides experimental evidence that, in the presence of dioxygen, hydrogen selenide (H_2_Se/HSe^−/^Se^2−^) damages DNA *in vitro*. Implication of free radicals in the damaging reaction is indicated by the protective effect afforded by mannitol. ESR analysis confirms the production of radicals from selenide.

Aqueous hydrogen selenide reacts with dissolved dioxygen, resulting in the formation of red colloidal selenium (Se_8_ ring), according to the following overall reaction:
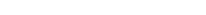
This reaction, which is completed in a few minutes in aerobic conditions, obeys a very complex kinetics [37] indicative of a free radical chain mechanism. Putative intermediate species include superoxide ion, hydrogen peroxide and polyselenides [37].

The closely related reaction between aqueous hydrogen sulfide and dioxygen is also believed to be autocatalytic [38], [39]. It is thought to start with the formation of sulfide radicals, followed by the production of polysulfide radicals which become active sites for oxygen chemisorption. Many additional reactions may take place, leading to the formation of radicals such as HSO_3_^•^, HSO_4_^•^, HS_n_SO_3_^•^ and maybe also ^•^OH.

Because of the chemical similarity between sulfur and selenium, several radicals, including selenium-containing radicals, are expected to be produced during oxidation of aqueous selenide by dioxygen. Thus, assignment of a particular radical to the breakage of DNA is difficult. ESR experiments are compatible with the formation of ^•^OH radicals upon selenide reaction with dioxygen. Inhibition by mannitol of the selenide-induced DNA breakage also suggests the production of ^•^OH. However, involvement in the DNA breaking reaction of some other undetermined radical cannot be formally excluded. In any case, our results exclude that superoxide anions account for DNA breakage. Indeed, mannitol, which does not quench superoxide anions, fully protects DNA in the nicking assay, and SOD addition has no effect in the same assay.

### Hydrogen Selenide Induces DSBs *in vivo*

The *in vitro* experiments with plasmid DNA raise the question as to whether the presence of hydrogen selenide originating from sodium selenide causes a breaking of phosphodiester bonds *in vivo*. If DSBs are indeed produced, proteins involved in the cellular response to DSBs (repair machinery and checkpoint proteins) should be important for cell survival in the presence of sodium selenide. Actually, in our genome-wide screen, we observed that the absence of a single protein participating in HR (the main and ubiquitous pathway for DSB repair) frequently caused hypersensitivity to sodium selenide. Another evidence for DSB induction upon sodium selenide treatment comes from cell-cycle analysis by flow cytometry. In the cellular context, a single DSB is enough to activate the G2/M checkpoint [40]. We indeed observed that exposure to Na_2_Se blocked the cells in G2/M. The percentage of blocked cells increased with the Na_2_Se concentration in the culture medium. The effect was amplified if HR was impaired, as shown with a *rad52* deletion mutant. DSB production could finally be evidenced *in vivo* using PFGE analysis. We thus propose that the production of hydrogen selenide from Na_2_Se induces DSBs, either by producing nearby SSBs on opposite strands or through replication of an SSB-containing DNA. Such a conclusion is reminiscent of that obtained with selenite [21], [22]. Because conversion of selenite to selenide is necessary to nick DNA *in vitro*, it is likely that selenite has to be reduced to selenide to exert its toxicity *in vivo*. This idea has already been developed elsewhere [9], [13].

### DSBs are the Main Cause of Cell Death in the Presence of Sodium Selenide

As mentioned above, different radicals are likely to be produced during the oxidation of selenide by O_2_. Therefore, several types of DNA damages may occur. Here, we obtain evidence that DNA fragmentation is the main cause of cell death. Firstly, sodium selenide addition is accompanied by a marked G2/M block. This behavior is expected with DSBs, not with DNA damages such as base oxidations. Secondly, the spectrum of genes associated with hypersensitivity to sodium selenide, as identified in our screen, resembles the spectra obtained with γ-ray [30] or Cpt treatments (Figure S3) which, each, predominantly induce DSBs. Thirdly, this spectrum is clearly different from the spectra obtained with several other physical or chemical genotoxic agents [31], [41]. Although we did not find genes of the BER pathway in the first 250 ranks, we cannot formally exclude an underestimation of the importance of this pathway in our approach, given the functional redundancies between proteins implicated in BER. Nevertheless, we believe that, in the case of the response to sodium selenide, the BER pathway is not as important as in the case of the response to other DNA-damaging agents such as UV. Indeed, a screen analysis of gene-disrupted yeast cells showed that disruption of *APN1* (a key gene in the BER pathway) confers hypersensitivity to UV [41]. In our screen, this gene is found at the 306th rank.

### Only a Few Genes of the Oxidative Stress Response Regulon are Involved in the Resistance to Sodium Selenide

Our data clarify the role of glutathione in selenium toxicity. *A priori*, glutathione may play two opposite roles in the selenide-induced oxidative stress [42]. On the one hand, glutathione is capable of scavenging various radicals including ^•^OH, and can therefore help the cell to face a selenide challenge. It can also repair proteins modified in the course of the selenide redox cycling. On the other hand, reduced glutathione can regenerate hydrogen selenide from elemental selenium [43], a compound itself produced by the oxidation of selenide by O_2_. Such a cycle is likely to accentuate the toxicity of hydrogen selenide. In our screening experiment in the presence of Na_2_Se, the *GSH1*, *GLR1*, *GRX1* and *GRX3* genes, involved in glutathione and glutaredoxin homeostasis, occupy ranks 1, 3, 7 and 31, respectively. Therefore, glutathione clearly emerges as an important player in the protection against sodium selenide. This suggests that the protective role of reduced glutathione predominates over its deleterious effect. Many other proteins belonging to the oxidative stress response, such as catalase, SOD, thioredoxins or glutathione peroxidases, are not determinant for the resistance to sodium selenide, since none of their genes ranks under 250 (Figure 1).

Our data pinpoint the importance of the DNA repair pathway in the response to sodium selenide exposure. Surprisingly, the genes involved in this pathway were not detected in a transcriptome analysis of selenite-stressed yeast cells [14]. Possibly, this absence reflects some differences between the cellular responses to selenide or selenite. Another explanation might be that, prior to a toxic challenge, corresponding gene products are already at sufficient concentrations in a cell to confer protection. Indeed, a study dealing with the identification of genes important for the survival of *S. cerevisiae* exposed to well known DNA-damaging agents (ionizing radiation, UV radiation, cisplatin, H_2_O_2_) concluded that transcriptions of most of the genes involved in protection against DNA damage were not stimulated in response to toxic doses of the damaging agent [44]. This may explain why the DNA repair pathway has been overlooked in gene expression profiling experiments.

## Supporting Information

Figure S1 **Strategy for quantitative analysis of a cell population using a two-step amplification of DNA barcodes.** Barcode regions are in gray. In the first PCR, one oligonucleotide hybridizes to a sequence beyond the barcode region (U1 for the upstream barcode, D1 for the downstream one) and the other one hybridizes inside the *kan*MX cassette (KU and KD, respectively). The second amplification is performed in the presence of fluorescent primers U2/D2 annealing inside the first amplicons, and in the presence of unlabeled U1/D1 primers. The PCR products (and thus the corresponding barcodes) can then quantified by microarray hybridization.
(EPS)

Figure S2 **Fitness defect analysis.** (**A**) Distribution of the relative fitness defect values (log_2_(s)) obtained with 4520 mutants of the systematic deletion collection. The observed distribution of fitness defect values (bins of size 0.01) was fitted to a Cauchy-Lorentz function by non-linear least squares regression. Part of the histogram and the fitted values are shown in the inset to illustrate the asymmetry of the results distribution, with an imbalance towards negative values (corresponding to strains that grew poorly in the presence of Na_2_Se). (**B**) Comparison of observed fitness defects obtained on pairs of strains that are deleted at the same locus. Data include 294 pairs of mutants for which there is an overlap of at least one nucleotide between the two corresponding deleted ORFs. An example is given at the top of the panel, where ORF YBR098W (*MMS4*) fully overlaps ORF YBR099C. The relative fitness defects observed for such pairs are represented as a scatter plot, arbitrarily assigning the mutated alleles to the X or the Y axis. The graph indicates high correlation between fitness values for each pair of mutants (non-parametric Kendall test, p<2.2·10^−16^).
(EPS)

Figure S3 **Correlation between the relative fitness defects associated with sodium selenide and Cpt treatments.** The data of the present study were compared with the fitness scores associated with Cpt treatment [1]. The comparison involves 4386 genes. The gray area corresponds to genes whose invalidation leads to fitness parameters smaller than −0.5 in the cases of the two toxic compounds. This comparison indicates a good correlation between the behaviors of the mutants in response to the two toxic agents (non-parametric Kendall test, p = 3.2·10^−14^). 1. Hillenmeyer ME, Fung E, Wildenhain J, Pierce SE, Hoon S, et al. (2008) The chemical genomic portrait of yeast: uncovering a phenotype for all genes. Science 320∶362–365.
(EPS)

Table S1 **Selenide sensitivity data on the systematic haploid deletion collection of** ***S. cerevisiae*** **strains.**
(XLS)

Table S2 **Weights used to filter the microarray data.**
(XLS)

Methods S1 (DOC)

Methods S2 (DOC)

## References

[1] Papp LV, Holmgren A, Khanna KK (2010). Selenium and selenoproteins in health and disease.. Antioxid Redox Signal.

[2] Brozmanová J, Mániková D, Vlcková V, Chovanec M (2010). Selenium: a double-edged sword for defense and offence in cancer.. Arch Toxicol.

[3] Ledesma MC, Jung-Hynes B, Schmit TL, Kumar R, Mukhtar H (2011). Selenium and vitamin E for prostate cancer: post-SELECT (Selenium and Vitamin E Cancer Prevention Trial) status.. Mol Med.

[4] Muecke R, Schomburg L, Buentzel J, Kisters K, Micke O (2010). Selenium or no selenium– that is the question in tumor patients: a new controversy.. Integr Cancer Ther.

[5] Selenius M, Rundlöf AK, Olm E, Fernandes AP, Björnstedt M (2010). Selenium and the selenoprotein thioredoxin reductase in the prevention, treatment and diagnostics of cancer.. Antioxid Redox Signal.

[6] Wu M, Kang MM, Schoene NW, Cheng WH (2010). Selenium compounds activate early barriers of tumorigenesis.. J Biol Chem.

[7] Jackson MI, Combs GF (2008). Selenium and anticarcinogenesis: underlying mechanisms.. Curr Opin Clin Nutr Metab Care.

[8] Seko Y, Imura N (1997). Active oxygen generation as a possible mechanism of selenium toxicity.. Biomed Environ Sci.

[9] Seko Y, Saito Y, Kitahara J, Imura N, Wendel A (1989). Active oxygen generation by the reaction of selenite with reduced glutathione in vitro.. Fourth international symposium on selenium in biology and medecine: Springer-Verlag, Heidelberg, Germany.

[10] Yan L, Spallholz JE (1993). Generation of reactive oxygen species from the reaction of selenium compounds with thiols and mammary tumor cells.. Biochem Pharmacol.

[11] Kitahara J, Seko Y, Imura N, Utsumi H, Hamada A, Sarkar B (1995). DNA strand breakage and lipid peroxidation as possible mechanisms of selenium toxicity.. Genetic response to metals.

[12] Kitahara J, Seko Y, Utsumi H, Hamada A, Imura N (1993). Possible role of active oxygen species in the toxic action of selenite.. Jpn J Toxicol Environ Health.

[13] Tarze A, Dauplais M, Grigoras I, Lazard M, Ha-Duong NT (2007). Extracellular production of hydrogen selenide accounts for thiol-assisted toxicity of selenite against *Saccharomyces cerevisiae*.. J Biol Chem.

[14] Salin H, Fardeau V, Piccini E, Lelandais G, Tanty V (2008). Structure and properties of transcriptional networks driving selenite stress response in yeasts.. BMC Genomics.

[15] Pinson B, Sagot I, Daignan-Fornier B (2000). Identification of genes affecting selenite toxicity and resistance in *Saccharomyces cerevisiae*.. Mol Microbiol.

[16] Seitomer E, Balar B, He D, Copeland PR, Kinzy TG (2008). Analysis of *Saccharomyces cerevisiae* null allele strains identifies a larger role for DNA damage versus oxidative stress pathways in growth inhibition by selenium.. Mol Nutr Food Res.

[17] Izquierdo A, Casas C, Herrero E (2010). Selenite-induced cell death in *Saccharomyces cerevisiae*: protective role of glutaredoxins.. Microbiology.

[18] Lewinska A, Bartosz G (2008). A role for yeast glutaredoxin genes in selenite-mediated oxidative stress.. Fungal Genet Biol.

[19] Anjaria KB, Madhvanath U (1988). Genotoxicity of selenite in diploid yeast.. Mutat Res.

[20] Mániková D, Vlasáková D, Loduhová J, Letavayová L, Vigasová D (2010). Investigations on the role of base excision repair and non-homologous end-joining pathways in sodium selenite-induced toxicity and mutagenicity in *Saccharomyces cerevisiae*.. Mutagenesis.

[21] Letavayová L, Vlasáková D, Spallholz JE, Brozmanová J, Chovanec M (2008). Toxicity and mutagenicity of selenium compounds in *Saccharomyces cerevisiae*.. Mutat Res.

[22] Letavayová L, Vlasáková D, Vlcková V, Brozmanová J, Chovanec M (2008). Rad52 has a role in the repair of sodium selenite-induced DNA damage in *Saccharomyces cerevisiae*.. Mutat Res.

[23] Nashef AS, Osuga DT, Feeney RE (1977). Determination of hydrogen sulfide with 5,5′-dithiobis-(2-nitrobenzoic acid), N-ethylmaleimide, and parachloromercuribenzoate.. Anal Biochem.

[24] Giaever G, Chu AM, Ni L, Connelly C, Riles L (2002). Functional profiling of the Saccharomyces cerevisiae genome.. Nature.

[25] Schwartz DC, Cantor CR (1984). Separation of yeast chromosome-sized DNAs by pulsed field gradient gel electrophoresis.. Cell.

[26] Nogi Y, Yano R, Nomura M (1991). Synthesis of large rRNAs by RNA polymerase II in mutants of *Saccharomyces cerevisiae* defective in RNA polymerase I. Proc Natl Acad Sci USA.

[27] Pietri S, Liebgott T, Fréjaville C, Tordo P, Culcasi M (1998). Nitrone spin traps and their pyrrolidine analogs in myocardial reperfusion injury: hemodynamic and ESR implications. Evidence for a cardioprotective phosphonate effect for 5-(diethoxyphosphoryl)-5-methyl-1-pyrroline *N*-oxide in rat hearts.. Eur J Biochem.

[28] Mazón G, Mimitou EP, Symington LS (2010). SnapShot: Homologous recombination in DNA double-strand break repair.. Cell.

[29] van Attikum H, Fritsch O, Gasser SM (2007). Distinct roles for SWR1 and INO80 chromatin remodeling complexes at chromosomal double-strand breaks.. EMBO J.

[30] Game JC, Birrell GW, Brown JA, Shibata T, Baccari C (2003). Use of a genome-wide approach to identify new genes that control resistance of *Saccharomyces cerevisiae* to ionizing radiation.. Radiat Res.

[31] Lee W, RP StOnge, Proctor M, Flaherty P, Jordan MI (2005). Genome-wide requirements for resistance to functionally distinct DNA-damaging agents.. PLoS Genet.

[32] Llorente B, Symington LS (2004). The Mre11 nuclease is not required for 5′ to 3′ resection at multiple HO-induced double-strand breaks.. Mol Cell Biol.

[33] Alabert C, Bianco JN, Pasero P (2009). Differential regulation of homologous recombination at DNA breaks and replication forks by the Mrc1 branch of the S-phase checkpoint.. EMBO J.

[34] Symington LS (2002). Role of *RAD52* epistasis group genes in homologous recombination and double-strand break repair.. Microbiol Mol Biol Rev.

[35] Hsieh HS, Ganther HE (1975). Acid-volatile selenium formation catalyzed by glutathione reductase.. Biochemistry.

[36] Frejaville C, Karoui H, Tuccio B, Le Moigne F, Culcasi M (1995). 5-(Diethoxyphosphoryl)-5-methyl-1-pyrroline *N*-oxide: a new efficient phosphorylated nitrone for the *in vitro* and *in vivo* spin trapping of oxygen-centered radicals.. J Med Chem.

[37] Nuttall KL, Allen FS (1984). Kinetics of the reaction between hydrogen selenide ion and oxygen.. Inorg Chim Acta.

[38] Tapley DW, Buettner GR, Shick JM (1999). Free radicals and chemiluminsecence as products of the spontaneous oxidation of sulfide in seawater, and their biological implications.. Biol Bull.

[39] Weres O, Tsao L, Chhatre RM (1985). Catalytic oxidation of aqueous hydrogen sulfide in the presence of sulfite.. Corrosion.

[40] Lee SE, Moore JK, Holmes A, Umezu K, Kolodner RD (1998). *Saccharomyces* Ku70, Mre11/Rad50 and RPA proteins regulate adaptation to G2/M arrest after DNA damage.. Cell.

[41] Hanway D, Chin JK, Xia G, Oshiro G, Winzeler EA (2002). Previously uncharacterized genes in the UV- and MMS-induced DNA damage response in yeast.. Proc Natl Acad Sci U S A.

[42] Shen H, Yang C, Liu J, Ong C (2000). Dual role of glutathione in selenite-induced oxidative stress and apoptosis in human hepatoma cells.. Free Radic Biol Med.

[43] Ganther HE (1971). Reduction of the selenotrisulfide derivative of glutathione to a persulfide analog by glutathione reductase.. Biochemistry.

[44] Birrell GW, Brown JA, Wu HI, Giaever G, Chu AM (2002). Transcriptional response of *Saccharomyces cerevisiae* to DNA-damaging agents does not identify the genes that protect against these agents.. Proc Natl Acad Sci U S A.

[45] Hillenmeyer ME, Fung E, Wildenhain J, Pierce SE, Hoon S (2008). The chemical genomic portrait of yeast: uncovering a phenotype for all genes.. Science.

