# Supplementary material for: Sodium Selenide Toxicity Is Mediated by O2-Dependent DNA Breaks
Source: PLoS One. 2012 May 7;7(5):e36343. doi: 10.1371/journal.pone.0036343 (PMC3346755; doi:10.1371/journal.pone.0036343)
Supplement: Methods S1 — (DOC) [file pone.0036343.s006.doc]

**Methods S1: Microarray estimation of growth rates**

The growth of individual strains was estimated from measurements done on custom barcode microarrays (Agilent G2509F - AMADID N°026035). Genomic DNA from Na_2_Se-challenged and control cells was extracted with phenol-chloroform from about 7.10^7^ cells broken by extensive vortexing in the presence of glass beads (425-600 nm size). DNA was used as template in two successive PCR reactions (Fig. S1). Upstream and downstream tag sequences were amplified in parallel. The oligonucleotides used in the first PCR reaction were: U1, GAT GTC CAC GAG GTC TCT and KU, AAG AAG AAC CTC AGT GGC for the upstream tags, and KD, GGA TCT TGC CAT CCT ATG and D1, CGG TGT CGG TCT CGT AG for the downstream tags. The obtained PCR products were verified by electrophoresis on an agarose gel and used in a labeling PCR reaction with the following Cy3 or Cy5 5'-labeled purified oligonucleotides: U2comp, GTC GAC CTG CAG CGT ACG for upstream barcodes and D2comp, CGA GCT CGA ATT CAT CGA T for downstream barcodes, and unlabeled U1 and D1. Only 15 cycles of amplification were used in the labeling step. The obtained PCR products were mixed and precipitated in the presence of linear acrylamide and of a mixture of complementary oligonucleotides in 4-fold molar excess (U2block, CGT ACG CTG CAG GTC GAC and D2block, ATC GAT GAA TTC GAG CTC G) to avoid binding of the fluorescently labeled oligonucleotides to the microarray probes. Hybridization was performed using the DIG Easy Hyb buffer (Roche Applied Science), at 24°C, overnight, in a rotating Agilent hybridization chamber. The slides were washed in decreasing concentrations of SSPE buffer (10 mM potassium phosphate (pH 7.4), 150 mM NaCl, 0.5 mM EDTA, 0.05% (w/v) Triton X100) down to 0.2 x SSPE, dried and treated immediately with the Agilent Stabilization and Drying Solution to avoid ozone-induced degradation of the Cy5 fluorophore. Scanning was performed on an Genepix 4200AL scanner and the images were analyzed using Axon Genepix Pro 7. The raw data were filtered by applying a weight to each data point. The weights (Table S2) were calculated on the basis of signal to noise ratio and variability of the signal for every barcode over 300 hybridization results and are close to 1 for oligonucleotide sequences that were reproducibly detected in most hybridizations (CM and CS, unpublished results). Filtered data coming from upstream or downstream barcodes were separately normalized using the Loess algorithm (R package marray included in Bioconductor [1]). Upstream and downstream barcode microarray values were averaged to obtain a single value for each mutant strain.

1. Gentleman RC, Carey VJ, Bates DM, Bolstad B, Dettling M, et al. (2004) Bioconductor: open software development for computational biology and bioinformatics. Genome Biol 5: R80.
