# Supplementary material for: Sodium Selenide Toxicity Is Mediated by O2-Dependent DNA Breaks
Source: PLoS One. 2012 May 7;7(5):e36343. doi: 10.1371/journal.pone.0036343 (PMC3346755; doi:10.1371/journal.pone.0036343)
Supplement: Methods S2 — (DOC) [file pone.0036343.s007.doc]

**Methods S2: Analysis of microarray data**

Normalized hybridization data were analyzed on the basis of several assumptions: (i) cells were grown under exponential growth conditions in rich medium and low cell density, which precludes competition effects. (ii) Most of the analyzed mutants have a growth rate close to the growth rate of the original wild-type strain. Thus, an estimation of the number of generations based on absorbance readings is reasonably accurate for the population as a whole. (iii) The Na_2_Se treatment globally affected the growth rate of all strains but had specific strong effects only on a relatively small number of strains.

To estimate the relative fitness defects of the mutants under Na_2_Se treatment, we used the ratios between the hybridization signals obtained for each mutant in a culture with Na_2_Se (culture A) and in a culture without Na_2_Se (culture B). The ratio between these signals (A/B) was deduced from the scan of a specific barcode microarray. Under the above assumptions, log_2_(A/B) = n_A_(d^Se^ – 1) – n_B_(d – 1) (equation (1)), where n_A_ and n_B_ are the number of generations accomplished by the wild-type strain in cultures A and B, respectively; d^Se^ is the growth defect of a given mutant in the presence of Na_2_Se, as compared to the wild-type (d^Se^ = t_wt_^Se^/t_mut_^Se^, where t_wt_^Se^ and t_mut_^Se^ are the generation times of the wild-type and of the mutant in the presence of Na_2_Se, respectively) and d is the growth defect of this mutant in the absence of Na_2_Se (d = t_wt_/t_mut_, where t_wt_ and t_mut_ are the generation times of the two compared strains in the absence of Na_2_Se). n_A_ and n_B_ can be deduced from the growth curves of the two cultures. To analyze our sets of data, we calculated a score, s, defined as s = d^Se^ – d + 1. With the wild-type, d = d^Se^ = 1 and the score is equal to 1. A slow-growing strain which has identical growth defects in the presence and in the absence of Na_2_Se (d^Se^ = d) also has a score of 1. A strain more sensitive to Na_2_Se than the wild-type (d^Se^ < d) has a score lower than 1. If n_A_ ≠ n_B_, equation (1) is not sufficient to calculate s. The growth defect of the mutant in the absence of Na_2_Se (d) must be known. This value was obtained for each mutant by comparing hybridization data from samples of the culture without Na_2_Se at t = 0 (sample C) and t = 16 h (sample D), using the equation log_2_(C/D) = n(1 – d), where C and D are the hybridization signals measured with the two samples and n is the number of generations accomplished by the wild-type during 16 h.

We calculated s values corresponding to four different conditions of Na_2_Se treatments (1 or 2 µM Na_2_Se treatment of cultures grown for either 16 or 27 h), with the Na_2_Se-free culture grown for 16 h as the reference. Because of too low signal-to-noise ratios when measuring hybridization signals, four s values could not always been obtained for each mutant strain. We computed the median of the s values when 3 or 4 culture conditions were available (Supplementary Table 1). The relative fitness defect was defined as the log_2_ value of s. With 267 mutants, we could not obtain data for more than two culture conditions. Results with such mutants were not used for further analyses.
